# Sequence analysis reveals mosaic genome of Aichi virus

**DOI:** 10.1186/1743-422X-8-390

**Published:** 2011-08-05

**Authors:** Xiaohong Han, Wen Zhang, Yanjun Xue, Shihe Shao

**Affiliations:** 1 School of Medical Science and Laboratory Medicine, Jiangsu University, 301 Xuefu Road, Zhenjiang, Jiangsu 212013, PR China

## Abstract

Aichi virus is a positive-sense and single-stranded RNA virus, which demonstrated to be related to diarrhea of Children. In the present study, phylogenetic and recombination analysis based on the Aichi virus complete genomes available in GenBank reveal a mosaic genome sequence [GenBank: FJ890523], of which the nt 261-852 region (the nt position was based on the aligned sequence file) shows close relationship with AB010145/Japan with 97.9% sequence identity, while the other genomic regions show close relationship with AY747174/German with 90.1% sequence identity. Our results will provide valuable hints for future research on Aichi virus diversity.

Aichi virus is a member of the *Kobuvirus *genus of the *Picornaviridae *family [1,2] and belongs to a positive-sense and single-stranded RNA virus. Its presence in fecal specimens of children suffering from diarrhea has been demonstrated in several Asian countries [3-6], in Brazil and German [7], in France [8] and in Tunisia [9]. Some reports showed the high level of seroprevalence in adults [7,10], suggesting the widespread exposure to Aichi virus during childhood.

The genome of Aichi virus contains 8,280 nucleotides and a poly(A) tail. The single large open reading frame (nt 713-8014 according to the strain AB010145) encodes a polyprotein of 2,432 amino acids that is cleaved into the typical picornavirus structural proteins VP0, VP3, VP1, and nonstructural proteins 2A, 2B, 2C, 3A, 3B, 3C and 3D [2,11]. Based on the phylogenetic analysis of 519-bp sequences at the 3C-3D (3CD) junction, Aichi viruses can be divided into two genotypes A and B with approximately 90% sequence homology [12]. Although only six complete genomes of Aichi virus were deposited in GenBank at present, mosaic genomes can be found in strains from different countries.

## Methods

### Sequences

The study sequences comprised six available complete genome sequences of Aichi virus from GenBank dated May 2011, including three Japan strains [GenBank: FJ890523, GenBank: NC_001918, GenBank: AB010145], one German strain [GenBank: AY747174], one Brazil strain [GenBank: DQ028632] and one China strain [GenBank: FJ890523]. Sequences were firstly screened to exclude patented and artificial mutants, and then aligned in the ClustalW program [13]. The alignment was manually adjusted for the correct reading frame. Sequences showing less than 1% divergence from each other were considered as the same. The remaining five genomes include two Japan strains, one German strain, one Brazil strain and one China strain.

### Phylogenetic Analysis and Recombination Detection

Before phylogenetic analysis, multiple-alignment was performed in the ClustalW program (http://www.clustal.org/). Phylogenetic trees were constructed using the neighbor-joining method and evaluated using the interior branch test method with Mega 4 software [14]. Percent bootstrap support was indicated at each node. GenBank accession no. was indicated at each branch. Detection of potential recombinant sequences, identification of potential parental sequences, and localization of possible recombination break points were determined using the Recombination Detection Program (RDP)[15], GENECONV [16], BOOTSCAN [17], MaxChi [18], CHIMAERA [19], and SISCAN [20] methods embedded in RDP3 [21]. A Multiple-comparison-corrected P-value cutoff of 0.05 was used throughout.

## Results and Discussion

**Figure 1:**
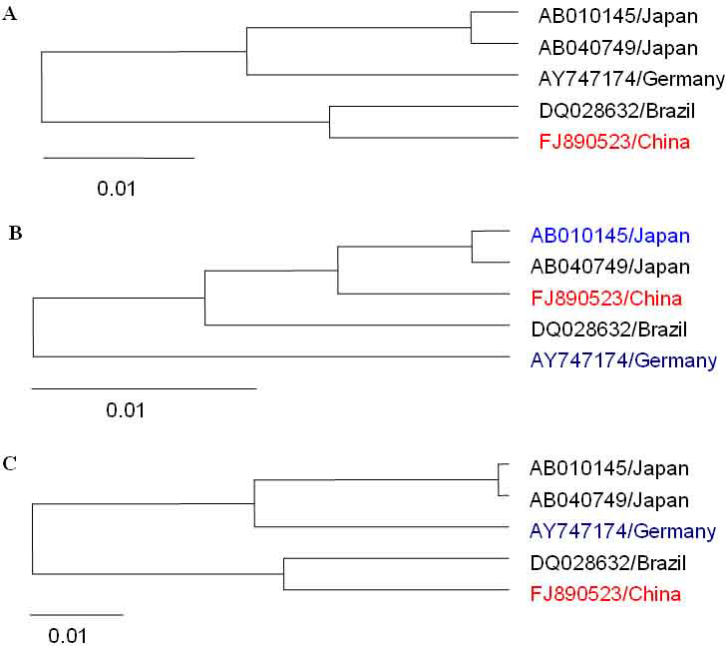
**Phylogenetic trees for the five Aichi virus strains**. Phylogenetic analysis were performed using the neighbor-joining method and evaluated using the interior branch test method with Mega 4 software. Values for various branches are percentages of the tree obtained from 1000 resamplings of the data. (A) Neighbor joining tree constructed basing on the complete genomes. (B) Neighbor joining tree constructed using the nt 261-852 region; (C) Neighbor joining tree constructed using the rest genomic region.

Based on the five complete genomes, a phylogenetic tree was constructed (Figure 1A). From the phylogenetic tree, we can see the five Aichi virus strains separated into two clusters. The two Japan Aichi virus strains [GenBank: AB010145, GenBank: AB040749] were closely related to the German strain [GenBank: AY747174] and formed into one cluster; while the Brazil [GenBank: DQ028632] and China Aichi virus strains [GenBank: FJ890523] clustered together, forming the other cluster.

**Figure 2:**
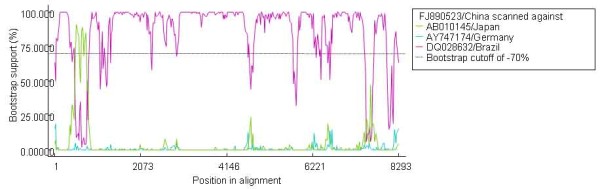
**Bootscan plots using sequence of the Chinese Aichi virus strain [GenBank: **FJ890523**] with Japanses Aichi virus strain [GenBank: **AB010145**] and German Aichi virus strain [GenBank: **Y747174**]**. The analysis was performed on the basis of pairwise distance, modeled with a window size 200, step size 20, and 100 Bootstrap replicates;

**Figure 3:**
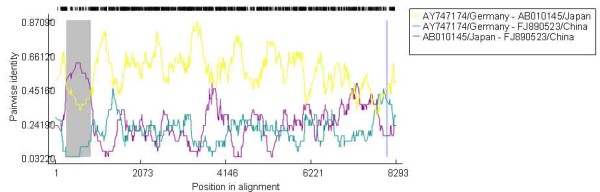
**RDP screenshots displaying the possible mosaic genome of [FJ890523/China]**. The y-axis indicates the pairwise identity that refers to the average pairwise sequence identity within a 30nt sliding window moved one nucleotide at a time. The area outlined in gray demarcates the potential mosaic regions.

Figure 2 indicated the Bootscan plots showing the likehood of recombinant sequence [GenBank: FJ890523], of which the nt 261-852 region shows close relationship with Japanese strain [GenBank:AB010145], while the other genomic regions show close relationship with the German strain [GenBank:AY747174]. The Bootscan results suggested that genome of Chinese Aichi virus strain [GenBank: FJ890523] was mosaic. This inference was confirmed by phylogenetic analysis, where two discordant phylogenetic relationship were showed in Figure 1B and 1C. Figure 1B indicated the tree constructed over the nt 261-852 region, where the Chinese strain [GenBank: FJ890523] was more closely related to the two Japan Aichi virus strains than to Brazil and German strains. Figure 1C indicated the tree established on the on the rest genomic region, which was similar to that constructed basing on the whole genome sequence, where the Chinese strain [GenBank: FJ890523] showed more closely to Brazil strain than to the other strains. Figure 3 indicated the pairwise identity between the Chinese strain [GenBank: FJ890523], German strain [GenBank: AY747174], and Japanese strain [GenBank:AB010145], which provide further evidence that mosaic-like genome exists in Aichi virus strain [GenBank: FJ890523].

Due to the limited numbers of Aichi virus sequence available at present days, the parental strains of the mosaic Aichi virus strain have not been identified in the present study. However, we think with the increasing number of Aichi virus genome, further study should be performed to elucidate whether accurate recombination event can happen between different Aichi virus strains. Because recombination is a relatively common phenomenon in positive-sense RNA viruses [22-24] and understanding recombination can be helpful in unravelling the evolution of pathogens and drug resistance.

## Conclusion

Taken together, this study reveals a mosaic genome sequence of Aichi virus [GenBank: FJ890523], of which the nt 261-852 region shows close relationship with strain of AB010145/Japan, while the other genomic regions show close relationship with German strain [GenBank:AY747174].

## Competing interests

The authors declare that they have no competing interests.

## Authors' contributions

XH and WZ conceived the study. All authors performed recombination analysis and critically reviewed and approved the final manuscript. WZ and XH wrote the paper. All authors read and approved the final manuscript
